# *Spitrobot-2* advances time-resolved cryo-trapping crystallography to under 25 ms

**DOI:** 10.1038/s42004-025-01784-9

**Published:** 2025-11-20

**Authors:** Maria Spiliopoulou, Caitlin E. Hatton, Martin Kollewe, Jan-Philipp Leimkohl, Hendrik Schikora, Friedjof Tellkamp, Pedram Mehrabi, Eike C. Schulz

**Affiliations:** 1 University Medical Center Hamburg-Eppendorf (UKE), Hamburg, Germany; 2 Institute for Nanostructure and Solid State Physics, Universität Hamburg, Hamburg, Germany; 3 Max-Planck-Institute for Structure and Dynamics of Matter, Hamburg, Germany

**Keywords:** Structural biology, Enzyme mechanisms

## Abstract

We previously introduced the *spitrobot*, a protein crystal plunging system that enables reaction quenching via cryo-trapping with a time resolution in the millisecond range. Here we present the next generation, *spitrobot-2*, as an integrated benchtop device. User-friendliness has been improved by semi-automatic sample exchange. Moreover, a fully automated shutter shields the liquid nitrogen from the humidified environment, improving sample integrity. Most importantly, the cryo-trapping delay time has been reduced to 23 ms, making *spitrobot-2* twice as fast as the previous generation. This further expands the number of target systems that can be addressed by cryo-trapping time-resolved crystallography. Using 12 crystal structures of three independent model systems, we demonstrate successful cryo-trapping via observation of conformational changes and ligand binding within 25 ms. These improvements increase the convenient access to cryo-trapping, time-resolved X-ray crystallography empowering the MX community with efficient tools to advance research in structural biology.

## Abstract (web summary)

Spitrobot is a protein crystal plunging system that enables reaction quenching through cryo-trapping with millisecond time resolution. Here, the authors present Spitrobot 2 as an integrated benchtop device with improved net sample preparation time and cryo-trapping delay time, increasing the number of target systems that can be studied using cryo-trapping time-resolved crystallography.

## Introduction

Time-resolved crystallography (TRX) is a powerful technique to study dynamic events and conformational responses of proteins while they carry out their function. In the past decade, it has undergone a resurgence, primarily triggered by the advent of X-ray free-electron laser (XFEL) sources and the emergence of serial data collection methods^1–3^. The successful application of serial crystallography at XFELs was quickly adopted at synchrotron facilities, which can provide equivalent data quality for sufficiently large crystals^4–7^. While serial synchrotron crystallography (SSX) at monochromatic MX beamlines cannot reach the ultra-fast time-scales that are accessible to XFELs, they are, however, the obvious choice when it comes to studying enzymatic mechanisms beyond the microsecond time domain. Their comparably wide distribution goes in hand with a lower competition for beamtime and thus enables a more democratic access, connected with the ability to study a larger number of model systems and develop novel methods that will benefit the field as a whole. The recent developments in time-resolved serial synchrotron crystallography (TR-SSX) have been the subject of a number of reviews^2,8–14^.

However, in spite of clear fundamental and practical advantages, as well as recent progress in simplifying the method, time-resolved crystallography still requires expert knowledge and thus often remains the niche of experts. Cryo-trapping crystallography is a convenient workaround that permits resolving metastable reaction intermediates by quickly quenching biochemical reactions in liquid nitrogen. Traditionally, slow enzymes with turnover times in the second’s domain or those with repetitive mechanisms have been addressed with cryo-trapping for decades^15,16^. While true TR-SSX experiments at room- or even physiological temperatures provide a much more complete insight into protein dynamics, and are not affected by vitrification artifacts^10,17–20^, these can be conveniently complemented by cryo-trapping experiments. Cryo-trapping allows for remote and decoupled sample preparation and data collection, requires fewer crystals for time-resolved studies, which favours challenging systems, e.g., hard to produce or unfavorable crystal size-to-diffraction ratios, and is compatible with low-brightness beamlines or even home sources, thus extending experimental capabilities to a wider range of setups and users. In addition to its conceptual simplicity, a major advantage of cryo-trapping is rooted in its compatibility with established high-throughput infrastructure and automated data-processing routines. However, a common problem in *manual* cryo-trapping lies in its limitation to macroscopic crystals, the comparably large time jitter associated with this, its comparably low reproducibility, especially if time-scales faster than ca. 30 s are aimed for. To address these limitations, we have recently developed the *spitrobot*, an automatic crystal plunger that enables time-resolved crystallography, via cryo-trapping, in the millisecond time domain^21^. Reactions are initiated by the *liquid application method for time-resolved applications* (LAMA), which permits in situ mixing with a minimal amount of substrate solution, while allowing for reaction initiation times in the millisecond time-domain. LAMA nozzles spray picoliter sized droplets of ligand solution onto protein crystals, which are exposed to X-rays or cryo-trapped after a pre-defined delay time^22^. The *spitrobot* is compatible with macroscopic crystals, micro-crystals, and canonical rotation, as well as serial data collection methods. In addition, the *spitrobot* is fully compatible with the SPINE standard (e.g., crystals are directly vitrified inside SPINE pucks), which directly connects cryo-trapping to the high-throughput infrastructure available at most synchrotrons. Another major advantage in cryo-trapping approaches lies in the ability to uncouple sample preparation from data collection, that is, users can prepare their samples well in advance to a beamtime and fully focus on either task.

For the growing user base interested in cryo-trapping crystallography using the *spitrobot*, we have updated the first generation prototype. *Spitrobot-2* is a fully integrated benchtop device with a minimal footprint, conveniently fitting into existing MX-laboratories. Like the first generation, *spitrobot-2* also maintains humidity and temperature conditions during reaction initiation via implementation of an environmental control system, and thereby also permits addressing long delay times^21^. Finally, we have substantially enhanced the user experience by improving the overall design and workflow of *spitrobot-2*. Most importantly, with further hardware improvements, we were able to reduce the minimum delay time by a factor of 2, now enabling cryo-trapping within less than 25 ms.

## Results and discussion

### An integrated benchtop device

**Fig. 1:**
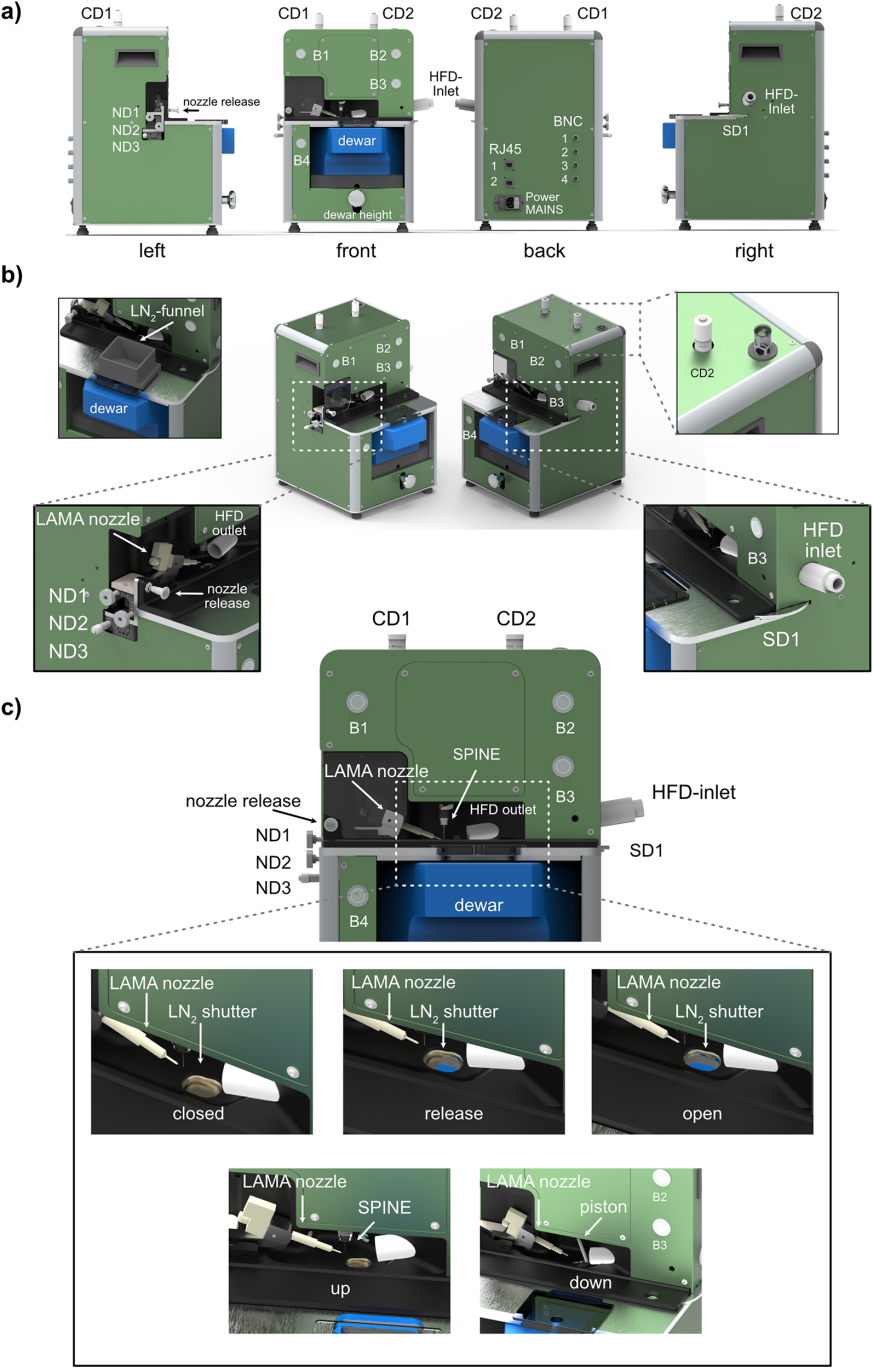
The redesigned *spitrobot-2.* **a** All side views of *spitrobot-2*. **b** 2-sided isometric view of *spitrobot-2* with closeups of the liquid nitrogen (LN_2_) funnel, the allen key lift, the nozzle dials (ND1-ND3), and the sample dial (SD). **c** The front-side of *spitrobot-2*, with closeups of the different liquid nitrogen shutter steps and the two main positions of the piston. Abbreviations: CD camera dial, ND nozzle dial, B button, SD sample dial, HFD humidity flow device.

In order to turn the *spitrobot* prototype into a user-friendly device, we aimed to generate a compact setup with minimal clutter that aids the user in the sample preparation process. For an overview incl. abbreviations, please refer to Fig. 1a, b. The footprint of *spitrobot-2* was substantially reduced, now comprising dimensions of W284 × H480 × D316 mm, with a weight of approximately 15 kg. Two handles on the sides allow for convenient relocation and enable use as a benchtop device. In contrast to the prototype, *spitrobot-2* permits triggering the plunging process from the main device itself, and therefore no longer depends on an external control box. The humidity flow device (HFD), as well as a control computer (for e.g., delay time setup, sample monitoring, automatic image capturing), remains separate.

The front panel comprises the two-hand-control safety switches that were formerly part of the control box (B1, B2). Pressing these buttons triggers reaction initiation and sample plunging according to the settings in the software interface. The two-hand-control solution prevents inadvertent interaction with the sample area during the plunging process and thereby prevents user injury. An additional button (B3) on the right side is used to reset the device in case of an error or as a light switch for sample illumination. To enable high-throughput sample preparation, samples are plunged directly into a SPINE puck. As in the prototype, a modified foam Dewar containing the SPINE puck is placed at the bottom, and its height is adjusted via an integrated LabJack. Sample exchange is executed via a sample exchange dial (SD1) that rotates the SPINE puck inside of the Dewar to access the next sample position (Supplementary Fig. 1). Next to the Dewar, on the lower left of the device, another button (B4) can be used to manually release the pin from the electromagnet on the piston. All buttons (B1–B4) are equipped with a color ring indicating the process or function status, e.g., red for error.

The sample viewing system consists of two cameras, aligned at an angle of 90° to each other. In *spitrobot-2* both cameras can be focused independently via two dials (CD1, CD2) on top of the device. To facilitate non-standard adjustments, such as precise camera centering, the device incorporates a built-in Allen key mechanism, which is automatically engaged and released by a spring-release mechanism, triggered by pressing the button. The LAMA nozzle for reaction initiation is mounted on the left side of *spitrobot-2*, and its precise orientation with respect to the sample position is controlled via three nozzle dials (ND1, ND2, ND3). The nozzle can be locked in a retracted position for sample mounting and quickly released into spraying position by the nozzle release latch. Different nozzle sizes are commercially available, enabling adjustment of the volume of the deposited substrate solution up to 3 nL/ms (Microdrop LLC, Norderstedt, Germany). The nozzle can be replaced by focusing optics, enabling optical excitation of the sample by a fibre-coupled light source or laser^23^. On the right side of *spitrobot-2* the HFD-nozzle is fed through to the sample position. On the back, there are two network ports, four BNC connections (e.g., for the LAMA trigger signal), as well as the main power supply and switch.

### A liquid nitrogen level indicator ensures sample quality

Sample quality is key to any TRX experiments, but for cryo-trapping TRX, this also includes the quality of the cryogen. The quality of the vitrified samples critically depends on the steepness of the temperature gradient and the purity of the liquid nitrogen, i.e., its level of water-ice contamination^24–28^. To support this, integrated temperature sensors warn the user or report an error if the level of liquid nitrogen drops too low, to avoid compromising the vitrification process and affecting the delay time. Liquid nitrogen refilling is possible via a safety funnel, which enables refilling while the Dewar remains in the sample preparation position. To insulate the liquid nitrogen from atmospheric air and the stream of highly humid, warm air from the HFD, *spitrobot-2* is equipped with a liquid nitrogen shutter window that only opens during the plunging period (Fig. 1c), blocking access to the cryogen at all other times. This limits liquid nitrogen evaporation and increases the cryogen life time in the Dewar, but more importantly, it reduces ice contamination, thereby improving sample quality. During the release stage, the window adopts a semi-open position, allowing for swift and reliable sample vitrification.

There is a clear influence of plunge freezing speeds and sample volumes on cooling rates and vitrification outcomes, which provides insights for further optimization. Previous studies on cryofixation emphasize that eliminating the cold gas layer above the liquid nitrogen during plunge freezing enhances cooling rates, thereby improving sample preservation^29^. Most of the cooling occurs in the cold gas layer above the cryogen during plunge cooling of protein crystals in liquid nitrogen^27^, and for plunge speeds of 1 ms^−1^, a 2 cm gas layer is sufficient to dominate the cooling rate^27,30^. *Spitrobot-2* is capable of efficient plunge freezing at 1.74 ms^−1^, further improving sample preparation.

### *Spitrobot-2* enables delay times of under 25 ms

Early vitrification experiments have provided valuable information on the cooling rates required to achieve successful vitrification. For pure water, these need to be as high as 10^6^ Ks^−1^, but this can be reduced by orders of magnitude by the presence of solutes^24,31^. Initial studies using liquid propane near its melting point as a cryogen achieved a peak cooling rate of about 370,000 Ks^−1^, determined by 25–75 μm thermocouples immersed at a rate of 2 ms^−1^ ^32^. Further research has highlighted significant differences in the cooling rates of different cryogenic media. In particular, the cooling rates in boiling liquid nitrogen have been reported to be about 50 times lower than those in, for example, 90 K ethane^33^, allowing vitrification without cryoprotectant. Although liquid nitrogen requires the use of cryo-protectants, the reduced safety concerns compared to flammable cryogens have made it the primary cryogen for X-ray crystallography^25,34^ and thus the primary choice for *spitrobot-2*.

The electro-pneumatic piston of the prototype has been replaced by a motorised solution, which in *spitrobot-2* drives the sample into a vial submerged in liquid nitrogen. In total, it is equipped with two linear motors (Faulhaber LM series), the first of which is used for the plunging process, while the second controls a shutter between the dispensing chamber and the Dewar. The plunger drives (Fig. 1c) the samples into the cryogen at a speed of 1.74 ms^−1^ (40 mm in 23 ms). To experimentally validate the minimum delay time that can be achieved with *spitrobot-2*, we measured the temperature evolution by plunging a 13 μm thermocouple into liquid nitrogen, with and without applying the humidity stream, as described previously^21^.

The metallic thermocouple sensor (nickel, type K) used for the cooling rate measurement has a diameter of 13 μm, while the crystals used in this study have diameters of around 20 μm. Based on their respective specific heat capacities (Table 1) we have determined that the equilibration time for the thermocouple as well as for protein crystals is in the micro-second domain, well below the experimentally measured quench time (Materials and Methods). Therefore, the thermocouple derived cooling times ares an appropriate estimate for the protein crystals used in this study.

**Table 1:** Thermodynamic parameters comparing the thermocouple and protein crystals

	thermocouple (nickel)	protein crystal (lysozyme)	
diameter of the sphere *d* (μm)	13	20	40
specific heat capacity ${c}_{p}\,(\frac{J}{kg\cdot K})$	444^73^		1750^58,74,75^
density $\rho \,(\frac{kg}{{m}^{3}})$	8908^76^		1240^77,78^
thermal conductivity *κ* (W/mK)	90^79^		0.42^80^
heat capacity of the sphere $C\,(\frac{nJ}{K})$	4.5	9.1	72.7
thermal diffusivity *α* (m^2^/s)	23 ⋅ 10^−6^		0.194 ⋅ 10^−6^
equilibration time *t*_*e*_ (μs)	0.55	155	620

**Fig. 2:**
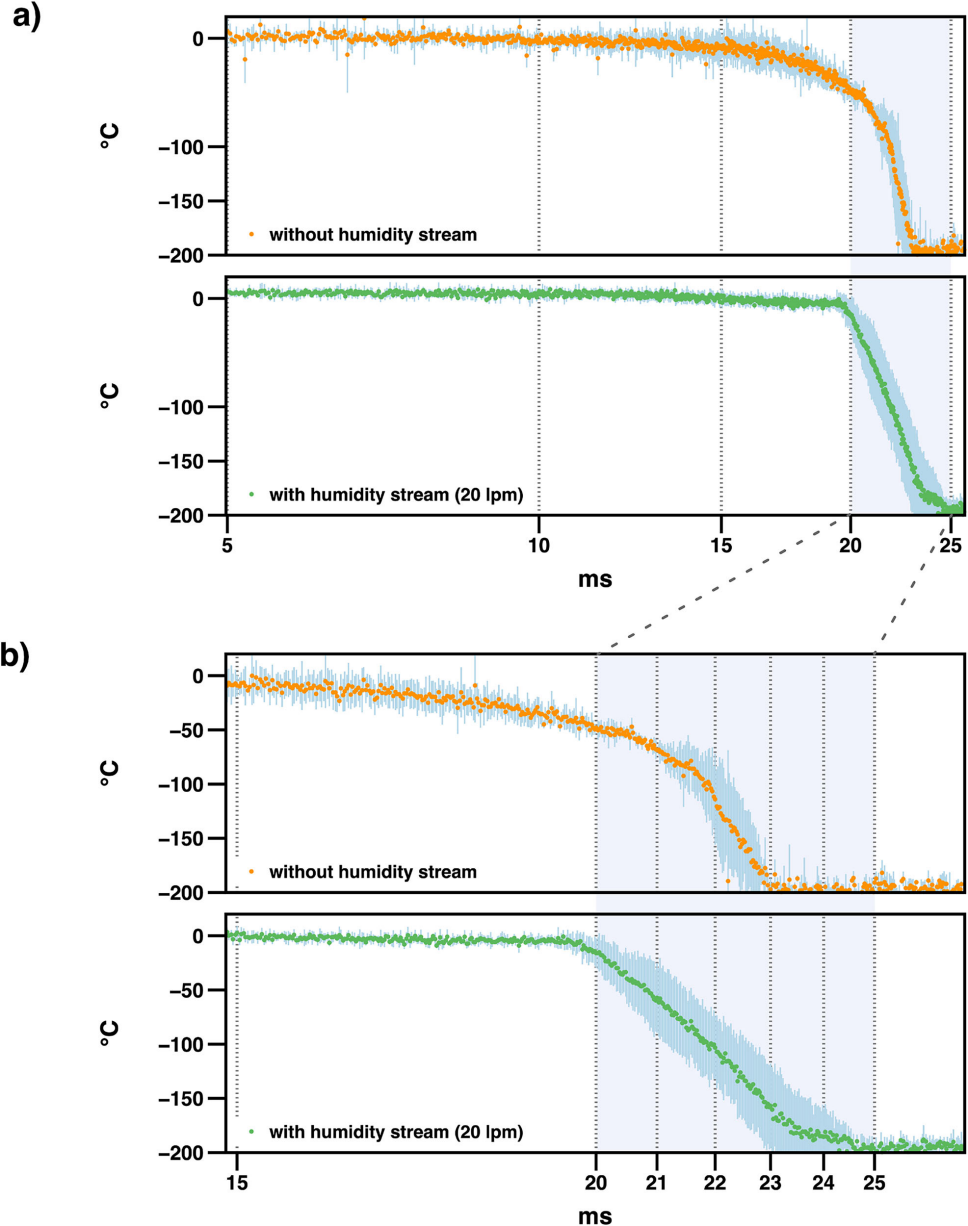
Average *spitrobot-2* vitrification time in liquid nitrogen. The temperature profile is shown without (orange, top-panel) and with (green, bottom panel) the humidity flow device, respectively. The standard deviation is shown as blue lines. Overall, the plunge process took 22.6 ± 0.4 and 23.6 ± 0.7 ms, respectively. **a** A global view of the average temperature evolution between 5 and 25 ms. Irrespective of the humidity flow device, the temperature drops to below −196 °C, within less than 25 ms. **b** A closeup of the vitrification time between 15 and 25 ms demonstrates that by application of the humidity flow device, the temperature remains stable until the sample enters the liquid nitrogen. The vitrification process in liquid nitrogen itself, then takes around 3.5 ms and thus is about 2 × faster than the vitrification time previously reported for the first generation.

As these data clearly show, *spitrobot-2* allows complete vitrification of the samples within 23 ms, which is about twice as fast as the prototype (Fig. 2a). For crystal sizes that are typically still useful for diffraction experiments at synchrotron sources (about 10–20 μm), this delay time now approaches the theoretical lower diffusion time limit for fast diffusing ligands such as glucose (15 ms)^10,22,35^. For practical purposes, it is important to consider that the ligand diffusion rate into protein crystals does not only depend on crystal packing and solvent channels, but also on the viscosity of the cryoprotectant. All these factors contribute to the adjustment of solvent viscosity and hydration levels that affect diffusion kinetics and ligand accessibility within the crystal lattice^36^. Notably, by using the HFD in *spitrobot-2* the cooling time measured by the thermocouple could be further reduced to 3.5 ms (Fig. 2 b). This equates to a cooling rate of approximately 57,000 Ks^−1^. While complete cooling to 77 K occurs on the order of a few milliseconds, the functionally relevant ’quench’ point is when the crystal passes below approximately 200 K, at which point enzymatic activity ceases^17,37–39^. For 20 μm crystals, this occurs within ca. 1–2 ms, and ca. 1 ms to reach the glass transition temperature, consistent with a recent study^40^. We anticipate that by selecting alternative cryogenic media, higher cooling rates may be possible with *spitrobot-2* in the future, potentially bypassing the need for cryo-protection.

A recent article describes instrumentation similar to *spitrobot-2*^40^. However, a side-by-side comparison of our system with the *mix-and-quench* approach of Indergaard et al. reveals some substantive differences:

*Spitrobot-2* achieves a minimum delay time of 23 ms, comprising the time from ligand application to complete vitrification. On the other hand, the *mix-and-quench* approach claims 8 ms delays by referring to the interval between ligand addition at the loop and immersion in liquid nitrogen, but excluding the vitrification time. Furthermore, although an 8 ms delay time has been reported, this has unfortunately not been validated by structural data, as there is no evidence of ligand binding below 150 ms^40^.

**Fig. 3:**
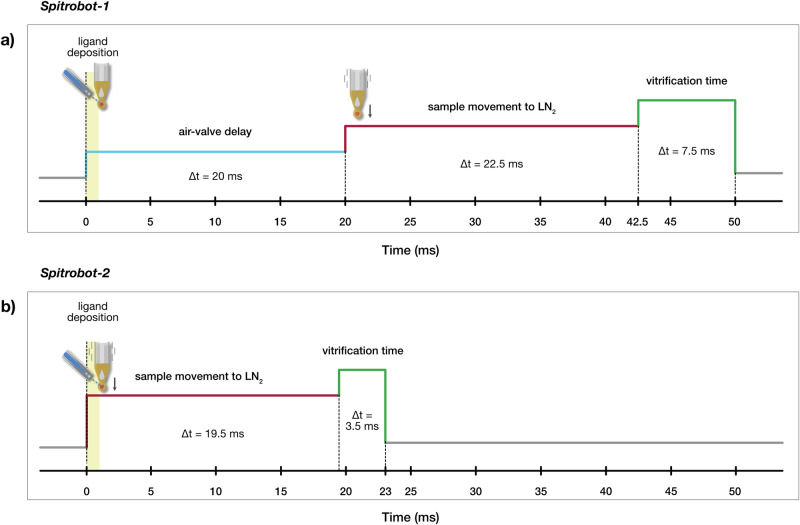
Comparison of the delay time between the *spitrobot* generations. **a** *Spitrobot-1*: The minimum total delay time is 50 ms: air-valve delay (20 ms), mechanical piston motion (22.5 ms), and vitrification time (7.5 ms); the ligand deposition (yellow) occurs during the air-valve delay. **b** *Spitrobot-2*: The minimum total delay time is 23 ms: electrical motor (plunger) motion (19.5 ms) and vitrification time (3.5 ms); the ligand deposition (yellow) occurs during plunger motion.

Moving towards the same objective and acknowledging the current needs in the field for more efficient time-resolved crystallography tools, *spitrobot-2* was developed to overcome some of the limitations observed in the previous generation (*spitrobot-1*), particularly regarding time delay. As described previously^21^, *spitrobot-1* is equipped with an air valve, and the motion of the piston is therefore delayed by 20 ms. *Spitrobot-2* is instead equipped with an electrical linear motor, enabling faster plunging of the sample to the liquid nitrogen (Fig. 3). This enables cryo-trapping experiments at delay times close to the theoretical diffusion limits.

### Sample preparation workflow

The original workflow of the *spitrobot* has been redesigned to provide a more user-friendly, streamlined experience that speeds up the entire sample preparation process. To start a sample preparation, the pre-cooled Dewar containing the SPINE puck is now placed on an integrated lifting mechanism. Once the Dewar has been inserted, precise positioning within the *spitrobot-2* is aided by a bright LED guiding beam which helps the user to align the puck with respect to the axis of the instrument’s rotational mechanism (Supplementary Fig. 1). Once in position, the Dewar is lifted by the labjack until it seals against the insulated and actively heated plate separating the Dewar from the upper part of the machine. This has two advantages: firstly, it helps to protect the operator during sample vitrification; secondly, it also greatly reduces ice formation within the liquid nitrogen as the Dewar is now isolated from both the atmosphere and the moisture stream. Thus, with these improvements the use time is approximately 30 min before refilling of liquid nitrogen is required. However, it is good common practice to top-up the liquid nitrogen after 3–5 samples have been plunged, to reduce sample variability. Sample access to the liquid nitrogen is only possible via the integrated shutter (*see above*). Next, the semi-manual sample dial on the right-hand side of the *spitrobot-2* is used to position the start vial in the immersion position. The alignment of the dispenser nozzle is supported by an integrated dual camera system (IDS imaging). Now the desired delay time can be set in the control software and the system is ready to start the plunging process by pressing the two-hand-control safety switches. Whilst retaining the two-handed safety switch solution implemented in the prototype, these have been integrated into the main unit so that *spitrobot-2* is no longer dependent on an external control box. Moreover, colour-coded signals integrated into the control buttons provide the user with immediate feedback about the plunging process, e.g., green and blue to let the operator know when to press or release buttons, or a white flash at the moment of sample excitation.

### Ligand binding events and conformational changes

To demonstrate the versatile applicability of *spitrobot-2* we demonstrate reaction initiation and fast delay times in three independent model systems, obtained by single crystal rotation data collection: *Streptomyces rubiginosus* xylose isomerase (XI), human insulin (HI), and bacteriophage T4 lysozyme (T4L).

#### Xylose isomerase ligand binding trapped in 25 ms

**Fig. 4:**
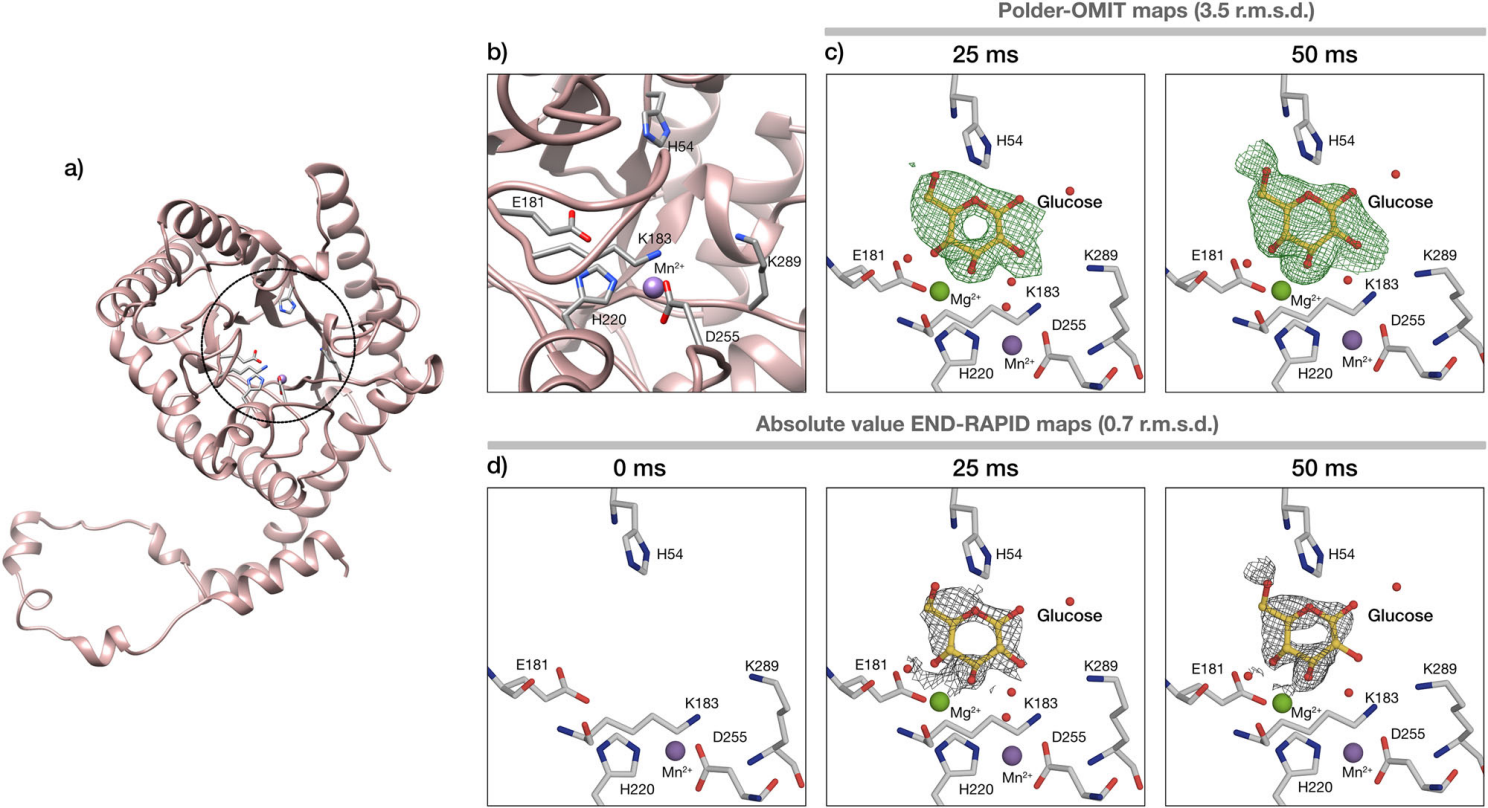
Glucose binding in the active site of XI. **a** Cartoon representation of the XI structure; the active site is indicated by a circle. **b** Closeup of the active site and surrounding residues. **c**, **d** Polder-OMIT maps and absolute value END-RAPID maps for glucose binding after 25 and 50 ms of soaking, respectively. Mg^2+^ and Mn^2+^ are represented as green and purple spheres correspondingly.

To allow direct comparison to the first generation of *spitrobot*, we used xylose isomerase (XI) as a model system. XI is of great commercial interest as it catalyses the reversible interconversion of *α*-D-glucose to *β*-D-fructose^41,42^. We have previously shown that glucose can bind to XI within 15 ms, which approximates theoretical diffusion times for crystals of this size^10,22,35^. To initiate substrate binding, we sprayed a 2 M glucose solution onto 20 μm XI crystals loaded on a micromesh before plunging them into liquid nitrogen with delay times of 25 ms and 50 ms, respectively. The difference density map in the active site shows that with *spitrobot-2* we can capture the diffusion of glucose after the minimum delay time of 25 ms (Fig. 4). This means that our current data confirm previous observations on glucose diffusion kinetics in protein crystals^22^. The onset of glucose binding was reported to occur at 15 ms using the same enzymatic system via SSX, and is consistent with the identification of a partially occupied state at 25 ms. In both studies, the ligand is observed in the active site, in the same conformation.

#### A pH jump triggers conformational changes in human insulin

Human insulin (HI) is a 5.8 kDa peptide hormone produced by *β*-pancreatic cells, which facilitates the absorption of glucose from the blood into tissues. It was one of the first proteins to be isolated and crystallographically studied^43–46^. In its active form, HI consists of 51 amino acids divided into two polypeptide chains: A and B (21 and 30 amino acids, respectively). The secondary structure of insulin shows two almost antiparallel *α*-helices in chain A and a *α*-helix followed by a turn and a *β*-strand in chain B^47^. After a century of crystallographic research, insulin has been identified as one of the most polymorphic proteins, displaying a range of molecular conformations, crystal forms, and binding capacities for metals and ligands^48^. Several cubic insulin crystal structures have been analysed under different conditions, such as crystals in 0.1 M sodium salt solutions with pH values ranging from 7.0 to 10.0^49^ and in 1 M Na_2_SO_4_ at pH 5.0 to 11.0^50^. It has previously been reported that cubic insulin crystals undergo a structural transition when the pH is lowered from 9.0 to 5.0^50^, while their crystal packing is not affected. The conformational change of glutamate at position 13 of chain B (GluB13) is accompanied by the binding of sulphate ions close to phenylalanine at position 1 of chain B (PheB1). Here, we aimed to induce this effect by a pH jump with *spitrobot-2*.

**Fig. 5:**
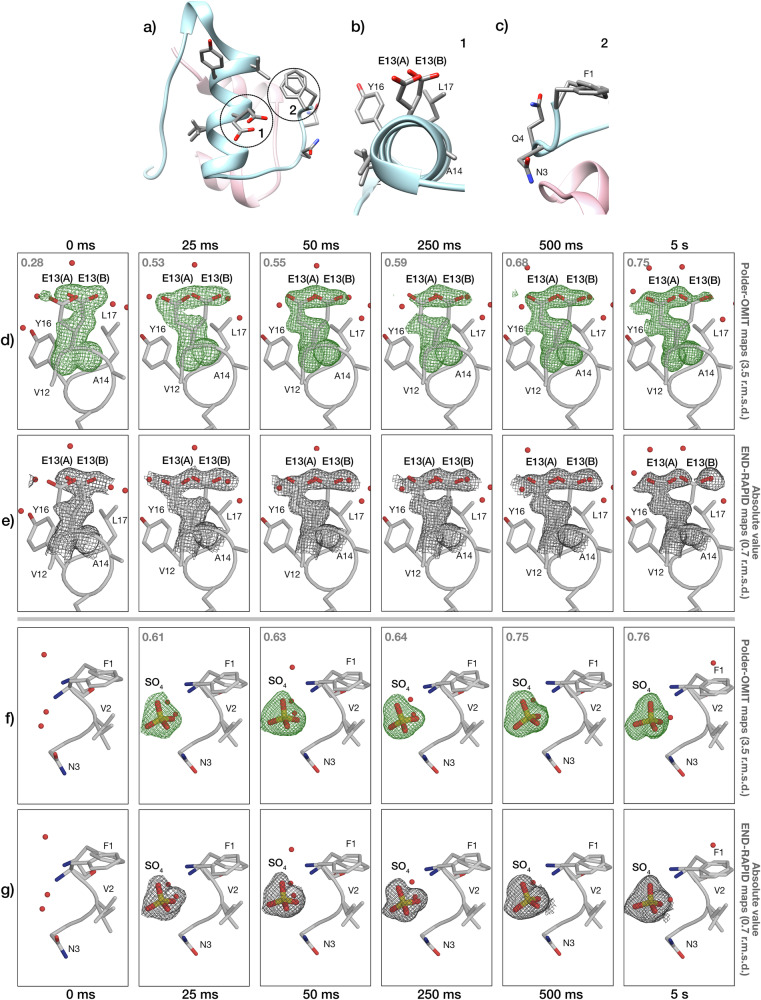
pH-jump effect on HI. **a** Cartoon representation of HI structure; the two chains are illustrated in pink (chain A) and blue (chain B), while the residues in the GluB13 region (1) and the SO_4_ binding region (2) are represented in sticks. **b** Closeup of GluB13 and surrounding residues for HI at pH 9.0. **c** Closeup of the SO_4_ binding region at pH 9.0 (apo state). **d**, **e** Polder-OMIT maps and absolute value END-RAPID maps for the effect of pH 4.5 on the GluB13 after 25 ms to 5 s; the occupancies of conformation A are indicated in grey. **f**, **g** Polder-OMIT maps and absolute value END-RAPID maps for the effect of pH 4.5 on SO_4_ binding after 25 ms to 5 s soaking; the occupancies for SO_4_ are indicated in grey.

To this end, we sprayed a low pH sulphate buffer (1 M CH_3_COONa, pH 4.5, 1 M Na_2_SO_4_, 15% (v/v) ethylene glycol) onto the insulin crystals and followed the motion of GluB13 and SO_4_ binding as a function of time. As can be clearly seen from the difference density maps, both the structural transition and the SO_4_ binding can be observed at delay times as short as 25 ms (Fig. 5; for refined occupancies for GluB13 and SO_4_ see Supplementary Table 2).

#### Backbone changes in bacteriophage T4 lysozyme (T4L)

**Fig. 6:**
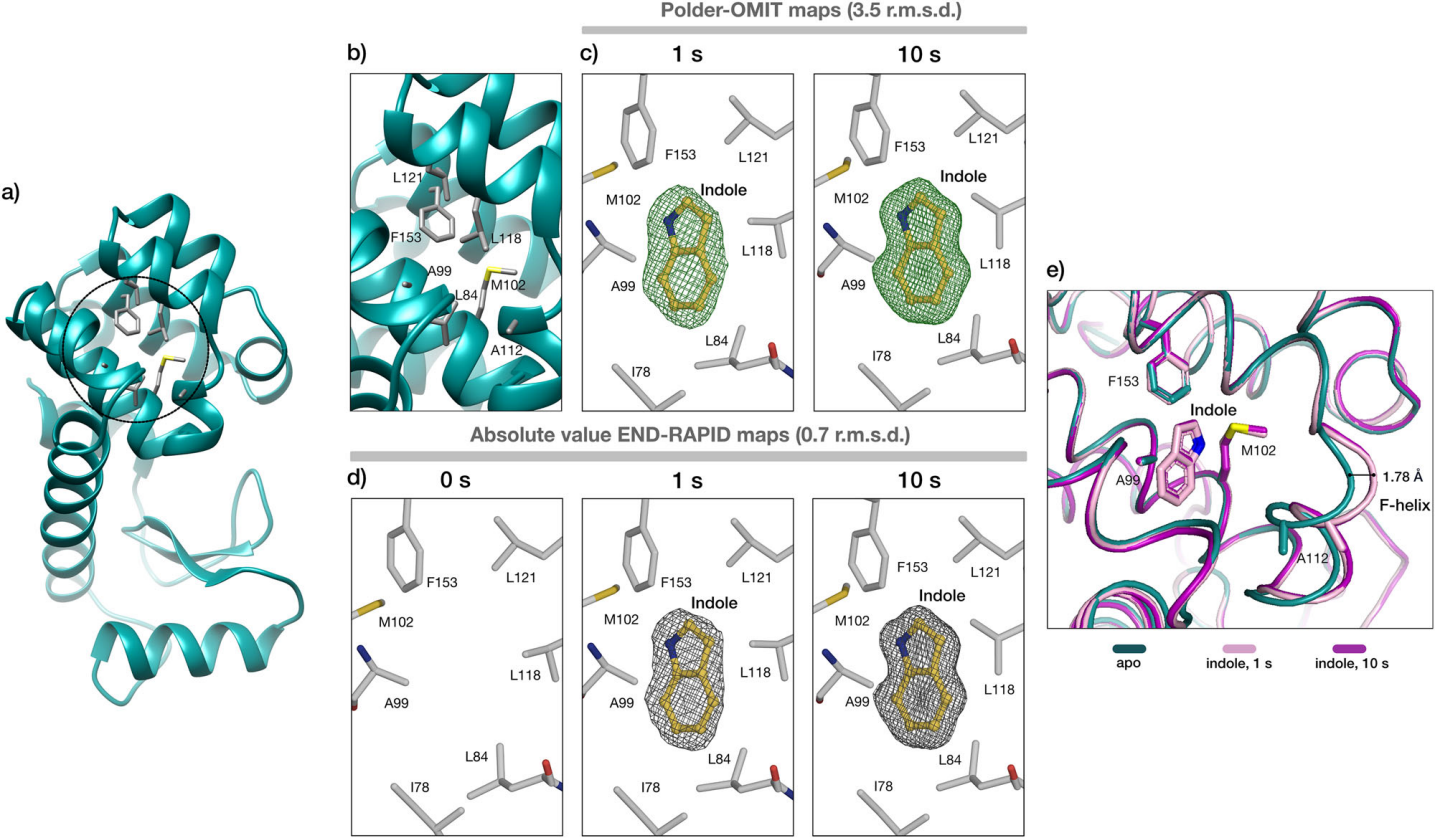
Indole binding in the non-polar cavity of T4L-L99A. **a** Cartoon representation of T4L-L99A structure; the cavity is indicated by a circle. **b** Closeup of the cavity and the surrounding residues. **c**, **d** Polder-OMIT maps and absolute value END-RAPID maps for indole binding after 1 s and 10 s of soaking time. **e** T4L-L99A cavity closeup. Ribbon representation of protein cavity in the absence (teal) and presence of indole after 1 s (pink) and 10 s (magenta) soaking time. The conformational change of the *F*-helix can be observed for the bound states of T4L, leading to a further opening of the cavity (1.78 Å) in the presence of the ligand.

The biological function of T4L is to hydrolyse the *β*-1,4 linkage between N-acetylmuramic acid and N-acetylglucosamine in bacterial peptidoglycan to aid cell lysis^51,52^. The L99A mutation of T4L forms an internal non-polar cavity of about 150 Å^3^, which allows the binding of various ligands after large conformational changes that open T4L to allow ligand binding^53,54^. As a result, this particular mutant serves as a model system for studying ligand binding at allosteric sites. Previous studies focusing on benzene binding pathways have proposed that structural rearrangements of the cavity surrounding the helices allow the ligand to enter the binding site^53–55^. To explore whether we could induce and capture larger conformational changes, we used indole as a ligand and observed structural changes after 1 s and 10 s, respectively. Our *spitrobot-2* cryo-trapping data clearly show that structural snapshots of the binding event can be obtained at delay times (1 s) inaccessible by manual methods (Fig. 6). In addition to indole binding, we clearly observe a conformational change in the backbone of the *F*-helix of T4L in the indole-bound states, which is shifted by about 1.8 Å compared to the apo state (Fig. 6e).

## Conclusions

*Spitrobot-2* is an integrated benchtop solution for cryo-trapping protein crystals with a delay time of less than 25 ms. It is suitable for monitoring ligand binding events as well as conformational changes of main and side chains. As the average enzyme has a turnover number of around 10 s^−1^^56^, a large number of systems should be amenable to cryo-trapping TRX, e.g., to track catalytic intermediates that are inaccessible by conventional methods. This is underlined by the fact that six highly different enzymes have now been studied by both generations of the *spitrobot*^21,23^)—testament to the device’s versatility and reliability. Combined with its small footprint and conceptual simplicity, these features should make *spitrobot-2* an attractive asset for MX labs wishing to standardize their vitrification process or explore cryo-trapping TRX. Its strength is further emphasized by its full compatibility with the high-throughput workflows found on most MX beamlines.

## Methods

### Temperature measurements

To assess the temperature evolution during the plunging process, we attached a 13 μm type K thermocouple (KFT-13-200-200, As One Corporation) to a canonical SPINE pin. The thermocouple was mounted on a SPINE pin at a distance of 22.0 mm from the base, within  ±1 mm at the sample position. This modified test sample was plunged into liquid nitrogen 3 times without, and 5 times with the humidity flow device, respectively. Temperature corresponding voltage measurements were recorded at 50 μs intervals. Furthermore, in order to reduce noise, we used a 100 μs low pass filter to connect the thermocouple to the oscilloscope (bandwidth: 350 MHz). Consequently, the readout lag is predominantly influenced by the low pass filter, but remains negligible in comparison to the overall vitrification time. In this context, vitrification time refers to the experimentally measure cooling time of the thermocouple.

#### Equilibration times

To clarify whether the thermocouple provides a sufficiently good approximation for the cooling of protein crystals, we determined the equilibration time of the thermocouple and the equilibration time of lysozyme crystals of different sizes. We consider a homogeneous spherical sample with thermal diffusivity *α*. The equilibration time *t*_*e*_ is defined as the time required for the temperature at the center of the sphere to reach 90% of the imposed temperature change at its boundary. Therefore,

$${t}_{e}\approx 0.3\cdot \frac{{r}^{2}}{\alpha }\,,$$

with *r* the radius of the sphere^57^. The thermal diffusivity *α* is given by the ratio of the thermal conductivity *κ* to the density of the material *ρ* and its specific heat capacity *c*_*p*_.

$$\alpha =\frac{\kappa }{\rho \cdot {c}_{p}},$$

The heat capacity *C* for the samples is given by

$$C=V\cdot \rho \cdot c,$$

wherein *V* is the volume of a sphere $(\frac{4}{3}\pi \,{r}^{3})$, *r* is the radius of the sphere, *ρ* is the density of the material, and *c*_*p*_ is the specific heat capacity. Based on the parameters in Table 1, the equilibration time within the 13 μm thermocouple is 0.55 μs, compared to 155 μs for 20 μm lysozyme crystals.

It is important to note that this estimate reflects the internal equilibration time within the sample, not the total quench time. This is set by the heat transfer from the surface of the sphere into the cryogen. Heat transfer from protein crystals during plunge-cooling into liquid nitrogen has been addressed in detail by Kriminski et al. They worked out that for sufficiently small samples (<500 μm), heat transfer is limited by the liquid boundary layer^58^. This is in agreement with earlier work, showing that internal heat conduction timescales are significantly shorter, thereby justifying the dominance of surface heat transfer in controlling the cooling dynamics^57^.

While the thermal diffusivity of lysozyme crystals is comparatively low with respect to the nickel thermocouple, the internal heat conduction is still well below the experimentally measured quench time. Therefore, the experimentally determined cooling of the nickel thermocouple provides a reliable benchmark for the effective quench rate using our plunge conditions.

### Protein purification and crystallization

#### Xylose isomerase

*Streptomyces rubiginosus* xylose isomerase in pET24a vector (Genscript), was expressed in BL21 DE3 E. coli cells, purified by affinity (Ni) and size exclusion chromatography with a final buffer of 0.05 M Tris pH 8.5, 0.15 M NaCl, with crystallization as reported in previous studies^22^. Briefly: 80 mg mL^−1^ XI was combined with an equal volume of crystallization buffer (35% (w/v) PEG 3350, 0.2 M LiSO_4_, and 0.01 M HEPES/NaOH, pH 7.5) via SpeedVac crystallization as previously described^59^.

#### Human insulin

Human insulin purchased from Roche (LOT: 70272900) was diluted in 0.05 M Na_2_HPO_4_, 0.001 M Na_2_EDTA, pH 11.0 (adjusted) to a final concentration of 30 mg mL^−1^. Batch crystallization was performed in a total volume of 160 μL by mixing equal volumes of HI and crystallization buffer consisting of 1.0 M NaH_2_PO_4_/K_2_HPO_4_, pH 9.0^60^.

#### Bacteriophage T4 lysozyme

T4-L99A mutant lysozyme gene was cloned in pET29b(+) vector (Genscript) and expressed in BL21 DE3 E. coli cells. The protein was purified by ion exchange and size exclusion chromatography with a final buffer of 0.05 M NaH_2_PO_4_/Na_2_HPO_4_, pH 5.5, 0.1 M NaCl, 0.002 M EDTA^61^. Batch crystallization was performed in a total volume of 20 μL by mixing equal volumes of protein at 22 mg mL^−1^ and crystallization buffer consisting of 4.0 M NaH_2_PO_4_/K_2_HPO_4_, pH 7.0, 0.1 M *1,6*-hexanediol, and 0.15 M NaCl (PDB ID: 3K2R).

### *Spitrobot-2* experiment parameters

Crystals of the three individual protein systems were used for the *spitrobot-2* experiments. The conditions and solutions used in each case are mentioned in Table 2.

**Table 2:** *Spitrobot-2* samples and environmental details

Sample	XI	HI	T4L-L99A
Crystal size (*μm*)	20	20	20
Temperature (°C)	25	25	25
Relative humidity (%)	95	95	95
Ligand	Glucose	Na_2_SO_4_	Indole
LAMA solution	0.01 M HEPES pH 7.5, 0.1 M LiSO_4_, 0.1 M MgCl_2_, 2 M glucose	1 M CH_3_COONa pH 4.5, 1 M Na_2_SO_4_, 15% (v/v) ethylene glycol	2.0 M NaH_2_PO_4_/K_2_HPO_4_, pH 7.0, 0.15 M NaCl, 0.1 M 1,6-hexanediol, 6% (v/v) DMSO, 0.05 M indole, 20% (v/v) glycerol

### X-ray data collection

Single crystal rotation datasets were collected at beamline P14 of PETRA-III (DESY, Hamburg) at the EMBL Hamburg outstation as well as at beamline ID30A-3 of ESRF (Grenoble, France). Data were collected on an Eiger2 CdTe 16 M detector at 12.7 keV and an Eiger1 X 4M detector at 12.81 keV, respectively for the aforementioned beamlines. Data collection parameters for each individual protein case are summarized in Supplementary Tables 1, 2 and 3 for XI, HI, and T4L-L99A, correspondingly.

### Data processing

Diffraction data were automatically processed with autoPROC using StarAniso^62–65^. Molecular replacement was conducted in Phaser using PDB-ID 8AWS, 1B18, and 4W51, respectively, as starting models for XI, HI, and T4L-L99A^66^. Structures were refined using iterative cycles of REFMAC or phenix.refine and coot^67–69^. Molecular images were generated in PyMol^70^.

Different map types were generated in order to examine lower occupancy features; Polder maps were generated to assess weak or ambiguous electron density by omitting both the region of interest and surrounding bulk solvent during map calculation using phenix.polder^71^. This approach enhances the visibility of low-occupancy features, such as ligands and alternative conformations, by reducing bulk-solvent model bias and preventing artificial density flattening. END-RAPID maps estimate local noise directly from experimental and model-derived errors^72^. These maps are presented on an absolute scale and visualized using a redefined noise threshold, enabling detection of weak but genuine electron density that may be obscured in conventional maps, and enables direct comparison of electron density features, since maps are on an absolute scale.

### Reporting summary

Further information on research design is available in the Nature Portfolio Reporting Summary linked to this article.

## Supplementary information

Supplemental Material

Description of Additional Supplementary Files

Supplementary Data 1

Reporting Summary
