# Supplementary material for: Spitrobot-2 advances time-resolved cryo-trapping crystallography to under 25 ms
Source: Commun Chem. 2025 Nov 20;8:363. doi: 10.1038/s42004-025-01784-9 (PMC12635289; doi:10.1038/s42004-025-01784-9)
Supplement: Supplementary file 1 — Supplemental Material [file 42004_2025_1784_MOESM1_ESM.pdf]

Supplementary Information

SPINE to UniPuck sample transfer

A common issue raised by external users of the prototype was the missing compatibility with UniPuck containers (https://smb.slac.stanford.edu/robosync/Universal_Puck/).

Although there is a commercial solution, the CombiPuck™ from MiTeGen, which enables sending samples stored in SPINE standard vials to beamlines that require UniPucks. For transferring samples between SPINE and UniPucks we provide details for in-house manufacturing of a similar transfer solution. We believe that this will alleviate previous concerns and simplify sample transfer between the different puck standards.

Supplementary figures

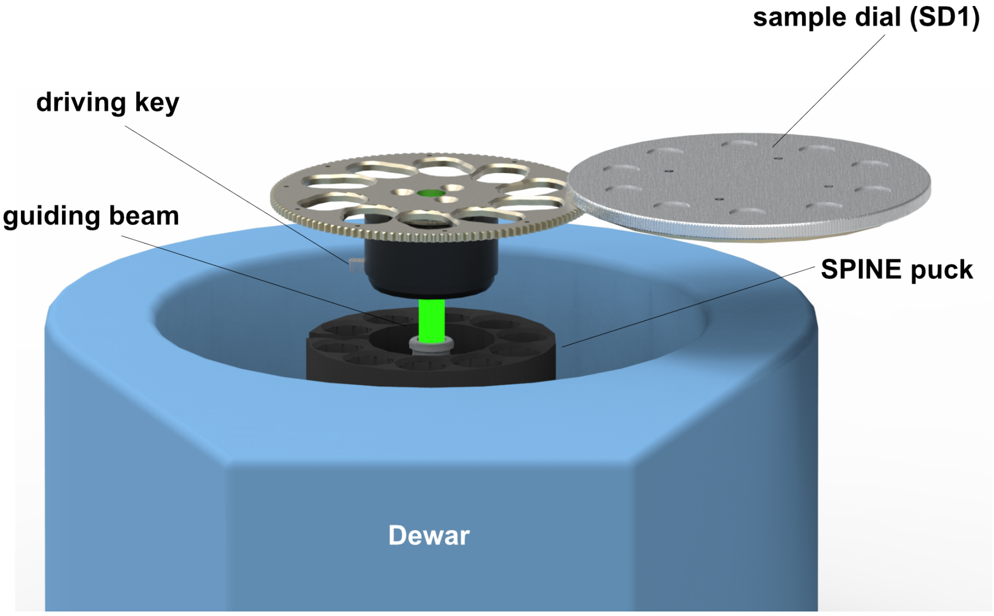

Supplementary Figure 1 Rendered cutaway view of the guiding beam and the sample-dial. Rendered illustration of the core components of the rotation mechanism and the guiding beam. The Dewar and SPINE puck are shown 20 mm below their actual position. Once the Dewar and puck are centered with the help of the guiding beam, they can be lifted using the in-built lab jack. The driving key then ensures a positive form-fitting connection between the puck and the rotation mechanism. Rotation of the sample dial (SD1) will be translated to a rotation of the SPINE puck enabling to manually switch between samples.

Data collection and refinement statistics

Supplementary Table 1 Data collection and refinement statistics of XI data. *Values in the highest resolution shell are shown in parentheses.*

**Sample**	**XI (apo)**	**XI (glucose, 25 ms)**	**XI (glucose, 50 ms)**
**PDB ID**	9R45	9R46	9R47
Data collection			
Source	P14, EMBL	P14, EMBL	P14, EMBL
Beam size (µm)	7 x 3	7 x 3	7 x 3
Flux (ph s^{-1})	9.254 x 10^{11}	2.037 x 10^{11}	2.037 x 10^{11}
Energy (keV)	12.7	12.7	12.7
Exposure time per frame (ms)	7.5	7.5	7.5
Number of images	3,600	10,800	3,600
Space group	$I222$	$I222$	$I222$
Unit cell parameters			
a, b, c (Å)	85.50, 93.72, 98.03	85.68, 93.76, 98.07	85.64, 93.92, 98.32
α, β, γ (°)	90	90	90
Resolution range (Å)	67.75-1.79 (2.02-1.79)	63.25-2.10 (2.18-2.10)	67.92-1.80 (1.95-1.80)
Total reflections	141,351 (6,541)	884,954 (39,157)	401,901 (17,336)
Unique reflections	20,717 (977)	23,420 (1692)	30,012 (1,501)
Mean I/σ(I)	5.9 (1.6)	24.6 (6.1)	8.4 (1.5)
Completeness (ellipsoidal)	88.3 (50.9)	100 (100)	93.8 (54.7)
Diffraction limits - ellipsoid fit (Å)			
along a*	2.67	-	1.96
along b*	1.79	-	1.93
along c*	1.97	-	1.80
Multiplicity	6.8 (6.7)	37.8 (24.0)	13.4 (11.5)
$CC_{1/2}$	0.983 (0.547)	0.999 (0.958)	0.996 (0.464)
Refinement			
Resolution range (Å)	67.74-1.79	64.52-2.10	49.16-1.80
Number of reflections	20,713	23,389	30,001
R_{work}	19.57	18.69	16.92
R_{free}	24.44	24.26	20.65
Occupancy - Glucose	-	0.53	0.56
Wilson B-factor ($Å^2$)	18.56	27.19	21.80
Mean B-factor ($Å^2$)			
Overall	20.70	29.49	23.75
Protein	20.31	29.12	22.97
Water	26.06	33.63	30.68
Other	41.85	33.17	29.41
Model quality			
RMS deviations			
Bond length (Å)	0.007	0.007	0.007
Bond angles (°)	0.874	0.850	0.834

Supplementary Table 2 Data collection and refinement statistics of HI data. *Values in the highest resolution shell are shown in parentheses.*

**Sample**	**HI (pH 9.0, apo)**	**HI (pH 4.5, 25 ms)**	**HI (pH 4.5, 50 ms)**	**HI (pH 4.5, 250 ms)**	**HI (pH 4.5, 500 ms)**	**HI (pH 4.5, 5 s)**
**PDB ID**	9R48	9R49	9R4A	9R4B	9R4C	9R4E
Data collection						
Source	ID30A-3, ESRF	ID30A-3, ESRF	ID30A-3, ESRF	ID30A-3, ESRF	ID30A-3, ESRF	ID30A-3, ESRF
Beam size (μm)	15 x 15	15 x 15	15 x 15	15 x 15	15 x 15	15 x 15
Flux (ph s^{-1})	5.22 x 10^{11}	5.18 x 10^{11}	5.23 x 10^{11}	5.16 x 10^{11}	5.23 x 10^{11}	5.20 x 10^{11}
Energy (keV)	12.8	12.8	12.8	12.8	12.8	12.8
Exposure time per frame (ms)	10	10	10	10	10	10
Number of images	900	900	900	900	900	900
Space Group	$I2_13$	$I2_13$	$I2_13$	$I2_13$	$I2_13$	$I2_13$
Unit cell parameters						
a (Å)	78.80	78.40	78.35	78.40	78.44	78.13
α (°)	90	90	90	90	90	90
Resolution range (Å)	39.40-1.65 (1.74-1.65)	39.20-1.64 (1.71-1.64)	39.17-1.44 (1.55-1.44)	39.20-1.49 (1.57-1.49)	39.22-1.41 (1.49-1.41)	39.07-1.29 (1.39-1.29)
Total reflections	71,234 (3,818)	59,591 (3,198)	72,904 (2,577)	115,848 (6,117)	111,825 (6,000)	90,466 (5,166)
Unique reflections	8,996 (450)	9,036 (452)	12,209 (610)	11,791 (590)	13,933 (697)	16,218 (811)
Mean I/σ(I)	9.9 (1.3)	6.4 (1.3)	8.2 (1.3)	8.2 (1.6)	8.9 (1.4)	6.5 (1.4)
Completeness (ellipsoidal)	94.1 (44.6)	93.3 (49.4)	92.4 (40.5)	94.2 (48.3)	94.1 (47.1)	91.4 (41.9)
Diffraction limits - ellipsoid fit (Å)						
along a*, b*, c*	1.68	1.66	1.50	1.52	1.44	1.36
Multiplicity	7.9 (8.5)	6.6 (7.1)	6.0 (4.2)	9.8 (10.4)	8.0 (8.6)	5.6 (6.4)
$CC_{1/2}$	0.996 (0.541)	0.982 (0.313)	0.994 (0.387)	0.994 (0.335)	0.997 (0.424)	0.992 (0.319)
Refinement						
Resolution range	39.43-1.65	39.23-1.64	39.20-1.45	39.23-1.50	39.25-1.41	39.10-1.30
Number of reflections	8,539	8,594	11,584	11,187	13,225	15,412
R_{work}	18.604	17.916	17.041	18.047	17.308	17.586
R_{free}	19.625	19.760	19.565	20.824	20.062	19.370
Occupancy - GluB13 (A/B)	0.28/0.72	0.53/0.47	0.55/0.45	0.59/0.41	0.68/0.32	0.75/0.25
Occupancy - SO_4	-	0.61	0.63	0.64	0.75	0.76
Wilson B-factor ($Å^2$)	16.41	10.66	12.29	12.13	10.75	10.16
Mean B-factor ($Å^2$)						
Overall	23.45	16.77	18.84	18.12	16.57	15.02
Protein	23.13	16.16	21.37	17.61	15.78	14.58
Water	34.80	29.50	31.20	31.50	30.30	26.20
Other	–	32.50	31.00	27.30	28.20	19.00
Model quality						
RMS deviations						
Bond length (Å)	0.0115	0.0124	0.0161	0.0136	0.0132	0.0130
Bond angles (°)	2.05	1.94	2.22	1.97	1.82	1.88

Supplementary Table 3 Data collection and refinement statistics of T4L-L99A data. *Values in the highest resolution shell are shown in parentheses.*

**Sample**	**T4L-L99A (apo)**	**T4L-L99A (indole, 1 s)**	**T4L-L99A (indole, 10 s)**
**PDB ID**	9R4F	9R4G	9R4H
Data collection			
Source	P14, EMBL	P14, EMBL	P14, EMBL
Beam size (µm)	50 x 50	50 x 50	30 x 30
Flux (ph s^{-1})	1.171 x 10^{12}	4.208 x 10^{11}	1.441 x 10^{12}
Energy (keV)	12.7	12.7	12.7
Exposure time per frame (ms)	7.5	7.5	7.5
Number of images	3,600	3,600	3,600
Space group	$P3_221$	$P3_221$	$P3_221$
Unit cell parameters			
a, b, c (Å)	60.01, 60.01, 96.25	60.17, 60.17, 97.22	60.24, 60.24, 97.71
α, β, γ (°)	90, 90, 120	90, 90, 120	90, 90, 120
Resolution range (Å)	52.05-1.50 (1.57-1.50)	52.11-1.90 (2.01-1.90)	52.17-1.49 (1.56-1.49)
Total reflections	550,310 (28,073)	214,047 (10,832)	591,509 (29,839)
Unique reflections	29,721 (1,383)	11,696 (586)	31,276 (1,548)
Mean I/σ(I)	12.4 (1.5)	8.4 (1.3)	15.9 (1.4)
Completeness (ellipsoidal)	94.5 (51.9)	87.4 (51.4)	95.1 (50.5)
Diffraction limits - ellipsoid fit (Å)			
along a* (0.90 a* - 0.45 b*)	1.50	1.83	1.52
along b*	1.50	1.83	1.52
along c*	1.58	2.51	1.49
Multiplicity	18.5 (20.3)	18.3 (18.5)	18.9 (19.3)
$CC_{1/2}$	0.998 (0.525)	0.992 (0.517)	0.999 (0.516)
Refinement			
Resolution range	52.10-1.50	52.17-1.90	51.22-1.49
Number of reflections	28,277	11,148	29,757
R_{work}	17.225	20.716	18.931
R_{free}	19.303	24.867	21.586
Wilson B-factor (Å^2)	16.01	19.47	18.80
Mean B-factor (Å^2)			
Overall	21.74	24.55	22.28
Protein	20.35	24.41	21.80
Water	22.20	26.64	29.70
Ligands	-	40.76	19.31
Other	-	-	26.22
Model quality			
RMS deviations			
Bond length (Å)	0.0103	0.0101	0.0110
Bond angles (°)	1.70	1.81	1.75
