# Supplementary material for: Spitrobot-2 advances time-resolved cryo-trapping crystallography to under 25 ms
Source: Commun Chem. 2025 Nov 20;8:363. doi: 10.1038/s42004-025-01784-9 (PMC12635289; doi:10.1038/s42004-025-01784-9)
Supplement: Supplementary file 2 — Description of Additional Supplementary Files [file 42004_2025_1784_MOESM2_ESM.pdf]

Description of Additional Supplementary Files

File name: Supplementary Data 1

Description: Source data for the graph in Fig2
